# Large Yellow Tea Extract Ameliorates Metabolic Syndrome by Suppressing Lipogenesis through SIRT6/SREBP1 Pathway and Modulating Microbiota in Leptin Receptor Knockout Rats

**DOI:** 10.3390/foods11111638

**Published:** 2022-06-01

**Authors:** Guohuo Wu, Xiaoyun Sun, Huijun Cheng, Shan Xu, Daxiang Li, Zhongwen Xie

**Affiliations:** 1 State Key Laboratory of Tea Plant Biology and Utilization, School of Tea and Food Sciences and Technology, Anhui Agricultural University, Hefei 230036, China; wgh1210@stu.ahau.edu.cn (G.W.); sun227206@126.com (X.S.); annongdaxushan@sina.com (S.X.); 2 College of Life Sciences, Anhui Agricultural University, Hefei 230036, China; c_huijun@126.com

**Keywords:** large yellow tea, *Lepr^−/−^* rats, hyperlipidemia, SIRT6/SREBP1 pathway, gut microbiota

## Abstract

Metabolic syndrome is a chronic metabolic disorder that has turned into a severe health problem worldwide. A previous study reported that large yellow tea exhibited better anti-diabetic and lipid-lowering effects than green tea. Nevertheless, the potential mechanisms are not yet understood. In this study, we examined the prevention effects and mechanisms of large yellow tea water extract (LWE) on metabolic syndrome using leptin receptor knockout (*Lepr^−/−^*) rats. Seven-week-old male *Lepr^−/−^* and wild type (WT) littermate rats were divided into *Lepr^−/−^* control group (KO) (n = 5), *Lepr^−/−^* with LWE-treated group (KL) (n = 5), WT control group (WT) (n = 6), and WT with LWE intervention group (WL) (n = 6). Then, the rats were administered water or LWE (700 mg/kg BW) daily by oral gavage for 24 weeks, respectively. The results showed that the administration of LWE significantly reduced the serum concentrations of random blood glucose, total cholesterol, triglyceride, and free fatty acids, and increased glucose tolerance in *Lepr^−/−^* rats. Moreover, LWE remarkably reduced hepatic lipid accumulation and alleviated fatty liver formation in *Lepr^−/−^* rats. A mechanistic study showed that LWE obviously activated SIRT6 and decreased the expression of key lipogenesis-related molecules SREBP1, FAS, and DGAT1 in the livers of *Lepr^−/−^* rats. Furthermore, LWE significantly improved microbiota dysbiosis via an increase in gut microbiota diversity and an abundance of the microbiota that produce short chain fatty acids (SCFAs), such as *Ruminococcaceae*, *Faecalibaculum*, *Intestinimonas,* and *Alistipes*. Finally, LWE supplementation increased the concentrations of SCFAs in the feces of *Lepr^−/−^* rats. These results revealed that LWE attenuated metabolic syndrome of *Lepr^−/−^* rats via the reduction of hepatic lipid synthesis through the SIRT6/SREBP1 pathway and the modulation of gut microbiota.

## 1. Introduction

Metabolic syndrome (MetS) is a chronic metabolic disorder that includes central obesity, hyperlipidemia, hypertension, and insulin resistance, and it has become a severe global health issue [1]. MetS displays a cluster of risk factors connected to type 2 diabetes (T2D) and cardiovascular disease (CVD) [2,3]. According to the International Diabetes Federation (IDF), more than a quarter of adults suffer from MetS [4,5]. It is believed that several factors contribute to the development of MetS, including genetics, diet, age, and lifestyle [6]. Moreover, researchers have identified a high correlation between gut microbiota and MetS [7]. Gut microbial dysbiosis frequently occurred in MetS individuals, who contained a larger abundance of opportunistic pathogens and a lower abundance of universal butyrate-producing bacteria in comparison with healthy controls [8,9]. This may lead to the reduction of SCFAs and the accumulation of injurious fermented products in the body [10]. There has been evidence that the microbiota alteration caused by phytochemicals from natural sources could be a new target for treating MetS. Compared with chemical synthetic drugs, a possible treatment of MetS using natural compounds derived from natural resources might be relatively inexpensive and have few side effects [11].

Tea prepared from the leaves and buds of the *Camellia sinensis* plant has been utilized in China as a medicine since ancient times and has become a widely consumed beverage throughout the world. Over the past three decades, researchers have studied the health benefits of tea, including weight loss, cardiovascular disease (CVD) and cancer prevention, and protection against neurodegenerative diseases [12,13,14,15]. However, the underlying molecular mechanisms are still unclear. If tea were capable of preventing or delaying the onset of these diseases, it would have a considerable impact on public health. There is mounting evidence that tea consumption alters the composition of microbes in the gut, which suggests that microbes may modulate the health benefits of tea consumption, such as lowering blood glucose levels and enhancing weight loss [16,17,18,19]. Yellow tea is a unique type of tea produced only in China. Unlike green tea, which is made of newly growing leaves and buds in the spring, large yellow tea is composed of one bud and three to six mature leaves, which are collected during summer or autumn from the tea plant. Our previous studies demonstrated that large yellow tea has remarkably hypoglycemic and hypolipidemic effects and alleviates glucose and lipid metabolism dysregulation in mice with high-fat diets. [20,21]. However, mice models are not appropriate for long-term investigation of MetS. Compared with mice models, rats are more physiologically similar to humans [22]. Currently, there are fewer studies using genetic rat models to investigate tea alleviating MetS. The *Lepr^−/−^* rat is a newly developed leptin receptor knockout rat model that has been demonstrated to be an appropriate animal model for studying MetS biomedically [23]. The chronic hyperglycemia of the *Lepr^−/−^* rat exhibited the advantages of long-term observation on the pathogenesis of MetS and diabetes. However, whether the large yellow tea alleviates MetS and hepatic steatosis in *Lepr^−/−^* rats has not been investigated. SIRT6 is an NAD^+^-dependent deacetylase that participates in the metabolic control of glucose and lipids in the liver [24]. Studies have demonstrated that SIRT6 could prevent fatty liver disease by reducing oxidative stress, endothelial dysfunction, and fibrosis [25,26,27,28]. Zhu et al. reported that Sirt6 regulates hepatic lipogenesis by inhibiting LXR and SREBP1 [29]. Previous research showed that green tea intervention reduced hepatic accumulation by suppressing the expression of SREBP1 [30]. However, it is not clear whether LWE can activate the SIRT6/SREBP-1 pathway to improve hepatic lipid metabolism. Additionally, the potential effect of gut microbiota modulation by large yellow tea in preventing MetS is still unclear.

The aim of this study was to test the hypothesis of whether LWE can efficiently prevent MetS and hepatic steatosis, and, if so, to determine the roles that the liver lipogenesis pathway and gut microbiota play in a *Lepr*^−/−^ rat model.

## 2. Materials and Methods

### 2.1. Analysis of Main Chemical Compounds of Large Yellow Tea

The large yellow tea was provided by Bao Er Zhong Xiu Tea Industry Co., Ltd. (Lu’an, China). The typical green tea, Huangshan Maofeng green tea (from Huangshan Maofeng Tea Group, Co., Ltd., Huangshan, China) was used for comparative analysis. Our previous methods were followed, with some minor modifications when preparing large yellow tea water extract (LWE) and Huangshan Maofeng green tea water extract (HWE) [21]. Briefly, 50 g of tea powder was boiled in 1000 mL of ultra-pure water and stirred for 30 min at 85 °C. Then, an ultrasonic extraction was conducted for 30 min at 75 °C using a power of 100 W on average. The yield ratio of lyophilized powder (based on weight/weight) was 34.3% for LWE and 30.8% for HWE, respectively.

Based upon our previous procedure [21], we analyzed the main catechins and caffeine with a Waters Ultra Performance Liquid Chromatography System (UPLC) equipped with a 600 controller, as well as a 2489 UV/Visible Detector. Quantitative analysis of amino acid content in the lyophilized powders of tea extracts was performed by using HITACHI (Tokyo, Japan) L-8900 automatic amino acid analyzer. In order to determine the polysaccharide content of tea water extracts, we used the phenol sulfuric acid method.

### 2.2. Animals Experimental Design

The *Lepr^−/−^* rats maintained in a Sprague–Dawley (SD) background were obtained from the Institute of Laboratory Animal Sciences, Chinese Academy of Medical Sciences (Beijing, China). The male *Lepr^−/−^* rats and wild type (WT) littermates were allowed to adapt to the specific pathogen-free (SPF) environment of the laboratory animal center at Anhui Agricultural University, which was maintained at standard temperature (22 ± 1 °C) and humidity level (50 ± 5%) during 12:12 h light and dark cycles. Water and food were freely available to all animals. At 7 weeks of age, WT rats were grouped into the WT control group (WT) (n = 6) and the WT with LWE intervention group (WL) (n = 6), while *Lepr^−/−^* rats were grouped into the *Lepr^−/−^* control group (KO) (n = 5) and the *Lepr^−/−^* with LWE treated group (KL) (n = 5). Rats in the LWE-treated groups were administered 700 mg/kg LWE daily by oral gavage for 24 weeks, and the control rats were given an equal volume of water at the same time. All procedures involving animals were approved by the Institutional Animal Care and Use Committee of the Anhui Agricultural University (ethical approval code: AHAU 2019-034).

The normal chow diet (12% fats, 20.6% proteins, and 67.4% carbohydrates, Table S1) was provided by Trophic Animal Feed High-Tech Co., Ltd. (Nantong, China). The daily consumption of food and water was monitored.

### 2.3. Serum Glucose and Oral Glucose Tolerance Test

Blood glucose was recorded in accordance with the previously described method using the Nova StatStrip Xpress Glucose CR Meter and its Glu-test Strips (Nova Biomedical, Waltham, UK) [21]. Oral glucose tolerance test (OGTT) was conducted during the 23-week treatment period. The fasting rats were given an oral administration of glucose (SIGMA, St. Louis, MO, USA) at a dose of 2 g per kilogram of body weight, and blood sample was taken from the tail vein of each animal, with blood glucose measurements taken at 0, 30, 60, 90, and 120 min before and after oral administration.

### 2.4. Serum Biochemical Parameters Measurement

To measure the blood lipid panel data, a total volume of 100 μL of blood was collected from the tail vein after fasting from 9:00 p.m. to 9:00 a.m. every 2 or 4 weeks. Serum triglycerides (TG), total cholesterol (T-CHO), high-density lipoprotein cholesterol (HDL-C), low-density lipoprotein cholesterol (LDL-C), alanine aminotransferase (ALT), and aspartate aminotransferase (AST) levels were measured using a kit from Nanjing Jiancheng Bioengineering Institute (Nanjing, China). Serum insulin, free fatty acid (FFA), and glycated serum protein (GSP) levels were measured via commercial enzyme-linked immunosorbent assay (ELISA) kits from Shanghai Jianglai Biotech (Shanghai, China). In order to calculate HOMA-IR, the homeostasis model assessment of insulin resistance, the following equation was used: HOMA-IR = fasting serum glucose (mmol/L) × fasting serum insulin (mIU/L)/22.5 [20].

### 2.5. Histological Analysis

The fixed liver and white adipose tissues were embedded in paraffin and then cut into 5-μm-thick sections, following the method described previously [31]. Liver paraffin sections were stained with hematoxylin-eosin (H&E) and Oil Red O (ORO) staining kits (Boster Bio, Wuhan, China). White adipose tissue paraffin sections were stained with H&E. The pictures were captured by microscope (LEICA DM500, Wetzlar, Germany) with a supporting camera (LEICA ICC50W, Wetzlar, Germany). Then, the areas (μm^2^) of the white adipocytes and the oil red area (μm^2^) of liver cells in sections were quantified by Image-J software (NIH, Bethesda, MD, USA), according to the previously reported method [21,32].

### 2.6. qPCR Analysis

As described previously [33,34], total RNA extraction from the liver tissues, reverse transcription PCR, and quantitative real-time PCR were conducted using RNA isolator, HiScript II 1st Strand cDNA Synthesis kit, and AceQ qPCR SYBR Green Master kit (Vazyme Biotech Co., Ltd., Nanjing, China), respectively. The primers sequences are provided in Table S2 in the Supplemental Materials.

### 2.7. Western Blot Analysis

The Western blot was performed in accordance with the previously described procedure [34]. Briefly, total proteins were extracted from the frozen liver samples using a 2 × SDS buffer. By using SDS-PAGE, equal amounts of denatured proteins were separated and were then transferred to PVDF membranes. After blocking the membranes with PBS-T solution of 5% skimmed milk for one hour, they were further incubated with sirtuin 6 (SIRT6), fatty acid synthase (FAS) (Cell Signaling Technology, Inc., Danvers, MA, USA), sterol regulatory element-binding protein 1 (SREBP 1), diacylglycerol-O-acyltransferase 1 (DGAT1) (Santa Cruz, CA, USA), and β-actin (Proteintech, Wuhan, China) antibody at 4 °C overnight. Next day, the membranes were incubated with secondary antibodies (Proteintech, Wuhan, China) for one hour at 25 °C. As part of the analysis, we used the ChemicDocTM MP Imaging System (Bio-Rad, Hercules, CA, USA) to detect the bands of proteins using enhanced chemiluminescence (ECL) reagent (Vazyme, Nanjing, China).

### 2.8. SCFAs Analysis in Feces

We measured fecal SCFAs by gas chromatography according to a previous protocol [35]. Assays for determination of fecal SCFAs were conducted with an Agilent 5977 gas chromatography system with a flame ionization detector (FID) and a high-resolution gas chromatography column (DB-WAX, 30 m × 0.25 mm × 0.25 µm). The contents of acetic acid, propionic acid, isobutyric acid, butyric acid, isovaleric acid, and valeric acid in each sample were measured based on external calibration curves. 

### 2.9. Gut Microbiota Analysis

Colon content samples were collected and transferred into DNase/RNase-free tubes and frozen in liquid nitrogen immediately when rats were sacrificed after 24-week intervention, then stored at -80 °C. Fast DNA SPIN Extraction Kit (MP Biomedicals, CA, USA) was used to extract the colon contents DNA. The 16S rRNA was amplified by PCR using forward 27F (5′-AGAGTTTGATCMTGGCTCAG-3′) and reverse 1492R (5′-ACCTTGTTACGACTT-3′) primers. The PCRs were performed as follows: 2 min of denaturation at 95 °C; 35 cycles of 30 s at 95 °C, 45 s for annealing at 60 °C, and 90 s for elongation at 72 °C; and a final extension step at 72 °C for 10 min. High-throughput sequencing was conducted using the PacBio RS II platform. Raw data were generated according to the protocol RS_ReadsOfInsert.1, and high-quality sequences were obtained using the QIIME package. To achieve a precise operational taxonomic unit (OTU) analysis, sequences containing errors have been removed below the threshold of 97% using UCLUST. In order to assign a taxonomy to each representative OTU sequence with an 80% confidence level, we used the Ribosomal Database Project II database. STAMP (Version 2.1.3) was used to identify phenotypes of gut microbes that differed significantly between different groups.

### 2.10. Statistical Analysis

Statistical analysis was performed by expressing the data as mean ± SEM. An unpaired 2-tailed Student’s t-test was conducted for comparison between the two groups, and a one-way ANOVA, followed by Tukey’s test, was conducted to compare the groups. The statistical significance was determined using *p* < 0.05.

## 3. Results

### 3.1. The Contents of Catechins, Caffeine, Amino Acids, and Polysaccharides in LWE 

The quantitative contents of catechins, caffeine, amino acids, and polysaccharides in LWE are shown in Table 1. The results indicated that LWE contains high levels of catechins (31.38 ± 1.44%), caffeine (11.12 ± 0.05%), and polysaccharides (18.80 ± 0.43%). Huangshan Maofeng green tea is a typical green tea in China. Here, we also measured the contents of catechins, caffeine, amino acids, and polysaccharides in HWE. Our data showed that the contents of catechins and EGCG in LWE were lower compared to HWE (*p* < 0.05). In addition, the total free amino acid content was also lower in LWE than in HWE. However, the concentrations of GCG, GC, caffeine, and polysaccharides in LWE were obviously higher than those of HWE (*p* < 0.05).

### 3.2. LWE Ameliorated Obesity Phenotype in Lepr^−/−^ Rats

**Figure 1:**
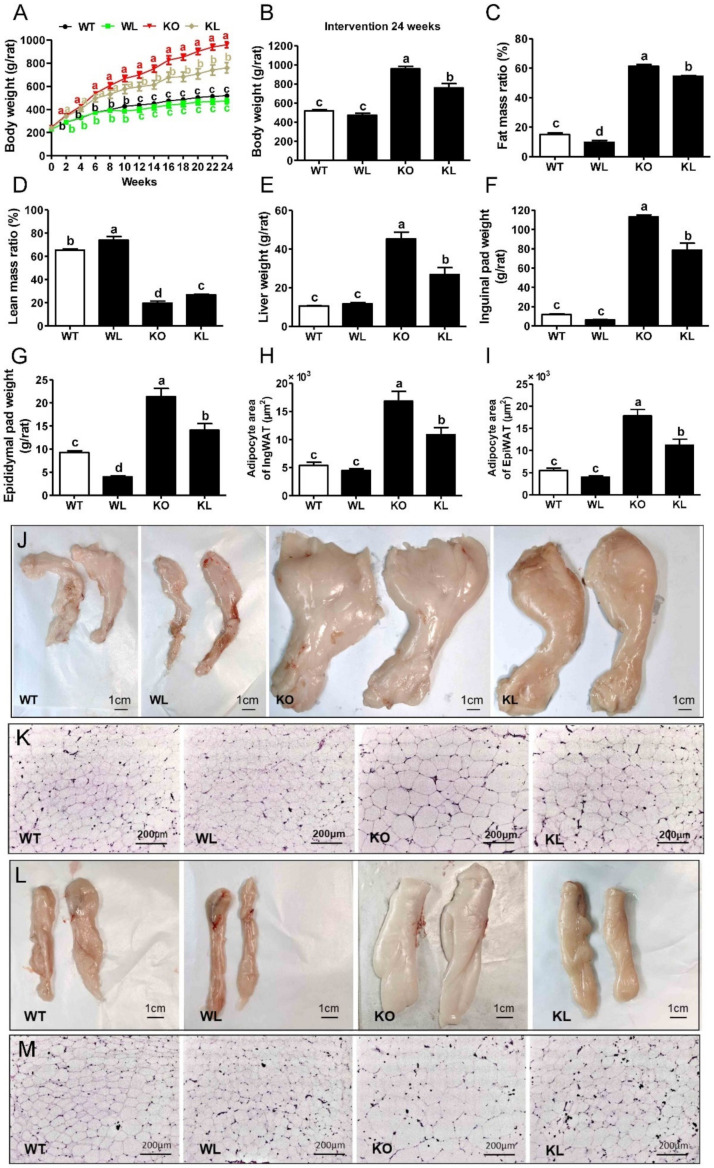
LWE ameliorated phenotype of obesity in *Lepr^−/−^* rats. LWE, water extract of large yellow tea; WT, wild type rat; WL, wild type rat with LWE intervention; KO, leptin receptor knockout rat; KL, leptin receptor knockout rat with LWE intervention. (**A**) Body weight curves, (**B**) body weight, (**C**) fat mass ratio, (**D**) lean mass ratio, (**E**) liver weight, (**F**) inguinal pad weight, and (**G**) epididymal pad weight of the rats were measured, respectively. The adipocyte area of (**H**) inguinal pad and (**I**) epididymal pad; the image of (**J**) inguinal pad and (**L**) epididymal pad in different groups of rats; the image of the inguinal pad (**K**) and epididymal pad (**M**) by H&E staining were at 100 ×magnification. Values are means ± SEM (n = 4–6). Columns with different letters are significantly different (*p* < 0.05).

Leptin receptor null rats display obesity, hyperphagia, glucose intolerance, and hyperlipidemia. According to Figure 1A,B, the body weight of *Lepr^−/−^* rats exhibited a rapid increase compared to the WT rats. However, LWE significantly decreased body weight in *Lepr^−/−^* rats at 14 weeks of intervention, and this preventive effect lasted until the end of the experiment. In addition, analyses of body mass composition showed that LWE supplementation greatly reduced fat mass gain and increased lean mass in *Lepr^−/−^* rats (Figure 1C,D). Moreover, food intake was elevated significantly in *Lepr^−/−^* rats (from 81.22 to 126.13 kcal/day/rat) compared with WT rats (*p* < 0.001). However, LWE treatment did not significantly alter food intake as compared to *Lepr^−/−^* rats.

After a 24-week experiment, the rats were sacrificed. The liver, inguinal, and epididymal pad tissues were collected and weighed (Figure 1E–G). The weights of the liver, inguinal, and epididymal adipose tissues were remarkably higher in the *Lepr^−/−^* group than in WT rats (*p* < 0.001). LWE supplementation significantly reduced the weights of the liver, inguinal, and epididymal adipose tissues compared to the *Lepr^−/−^* control group. Additionally, compared with the WT rats, the adipocytes in the inguinal pad and epididymal pad of the *Lepr^−/−^* rats were obviously enlarged (Figure 1H,I). After intervention with LWE, the areas of adipocytes were significantly decreased in the inguinal pad and epididymal pad of *Lepr^−/−^* rats.

### 3.3. LWE Improved Lipid Profiles in Serum and Liver of Lepr^−/−^ Rats

**Figure 2:**
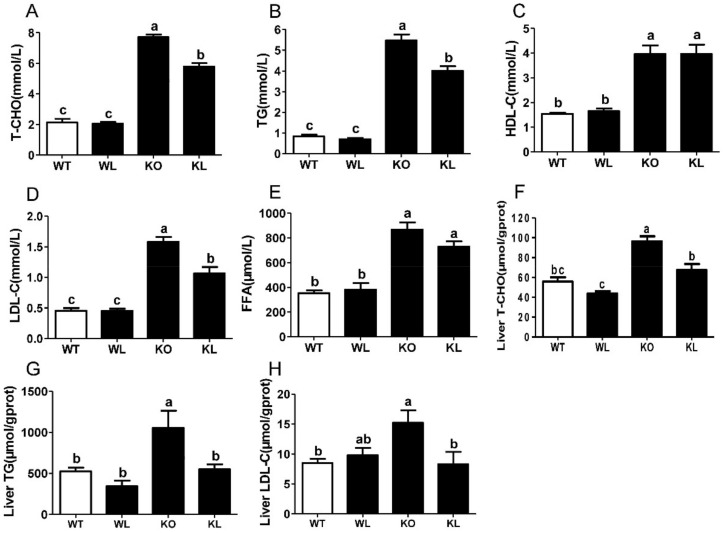
Biochemical indexes of serum and liver in each group of rats. (**A**) Serum total cholesterol; (**B**) serum triglyceride; (**C**) serum high-density lipoprotein cholesterol; (**D**) serum low-density lipoprotein cholesterol; (**E**) serum free fatty acids; (**F**) liver total cholesterol; (**G**) liver triglyceride; (**H**) liver low-density lipoprotein cholesterol; Values are means ± SEM (n = 4–6). Columns with different letters are significantly different (*p* < 0.05).

The concentrations of serum T-CHO, TG, HDL-C, and LDL-C were determined. A significant increase in serum T-CHO, TG, HDL-C, and LDL-C levels were found in *Lepr^−/−^* rats compared to WT rats. While LWE treatment significantly reduced the levels of serum T-CHO, TG, and LDL-C in *Lepr^−/−^* rats (*p* < 0.05) (Figure 2A,B,D), but the levels of HDL were not significantly different between the *Lepr^−/−^* group and *Lepr^−/−^* + LWE group (Figure 2C). Measurements of FFA levels showed that LWE effectively prevents the elevation of FFA levels in *Lepr^−/−^* rats (*p* < 0.05) (Figure 2E). In addition, the levels of liver lipids including T-CHO, TG, and LDL-C were examined. The results indicated a significant increase in T-CHO, TG, and LDL-C levels in the livers of the *Lepr^−/−^* group compared to the WT group (Figure 2F–H). The LWE intervention obviously decreased the concentrations of TG, TC, and LDL-C in the livers of *Lepr^−/−^* rats (*p* < 0.05). The results showed that LWE significantly improved the serum and liver lipid profiles to attenuate the MetS of *Lepr^−/−^* rats.

### 3.4. LWE Improved Glucose Tolerance in Lepr^−/−^ Rats

**Figure 3:**
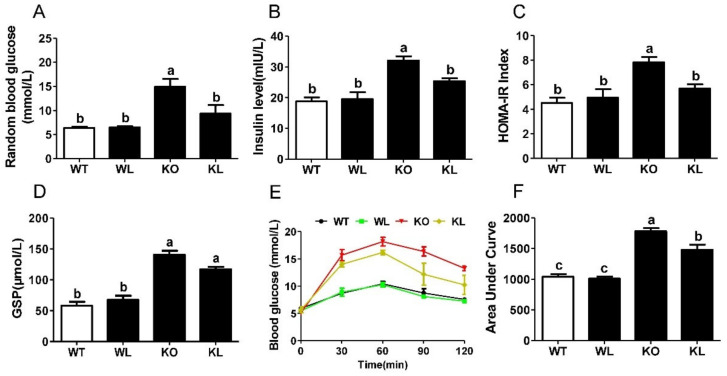
LWE decreased blood glucose and improved glucose tolerance in *Lepr^−/−^* rats. (**A**) Random blood glucose, (**B**) fasting serum insulin, (**C**) HOMA-IR, (**D**) glycated serum protein level was measured; (**E**) oral glucose tolerance test; (**F**) area under the curve of OGTT. Values are means ± SEM (n = 4–6). Columns with different letters are significantly different (*p* < 0.05).

Throughout the entire rat experimentation period, we measured blood glucose level dynamically. The *Lepr^−/−^* rats showed elevation of random blood glucose and serum insulin levels (*p* < 0.05) (Figure 3A,B). The consumption of LWE obviously decreased the random blood glucose and serum insulin levels of *Lepr^−/−^* rats (*p* < 0.05) (Figure 3A,B). Fasting glucose levels in the *Lepr^−/−^* rats had no statistical significance compared to the WT rats (Figure S3). Insulin sensitivity was evaluated using the HOMA-IR index. Our data showed that LWE intervention significantly improved the HOMA-IR index in *Lepr^−/−^* rats (Figure 3C). Moreover, the glycated serum protein (GSP) level in the *Lepr^−/−^* rats was obviously higher than that in WT rats (*p* < 0.01), while LWE prevented the increase of GSP level in *Lepr^−/−^* rats (Figure 3D). An oral glucose tolerance test (OGTT) was conducted in order to assess the ability of the test animals to dispose of the glucose load. Compared with WT rats, *Lepr^−/−^* rats demonstrated significantly elevated blood glucose levels before and after glucose loading (Figure 3E). An increase in the AUC of the OGTT was observed in *Lepr^−/−^* rats, suggesting severe glucose intolerance (Figure 3F). LWE treatment resulted in a reduction in AUC in *Lepr^−/−^* rats (*p* < 0.05). These results indicated that LWE improved insulin resistance and glucose tolerance in *Lepr^−/−^* rats.

### 3.5. LWE Alleviated Fatty Liver Formation in Lepr^−/−^ Rats

**Figure 4:**
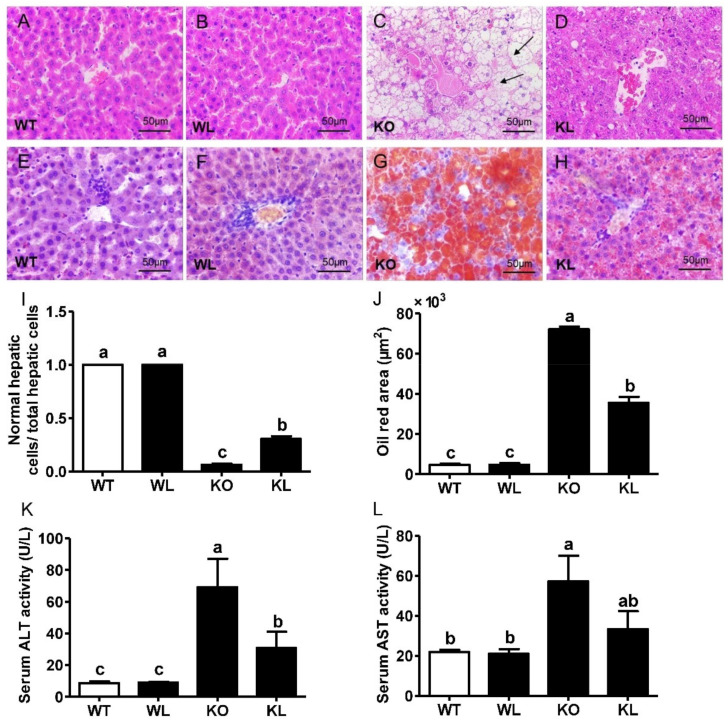
LWE ameliorated fatty liver formation in *Lepr^−/−^* rats. (**A**–**D**) Liver HE staining; (**E**–**H**) liver ORO staining; (**I**) ratio of normal hepatic cells to total liver cells (5 fields of view were randomly selected from each tissue section for statistical purposes); (**J**) oil red area of the liver; (**K**) serum ALT activity; (**L**) serum AST activity (n = 4–6). Columns with different letters are significantly different (*p* < 0.05). black arrows present hepatic fatty vacuoles.

The liver is a central organ for the maintenance of systemic lipids and glucose homeostasis. A fatty liver is often associated with obesity. Our data showed that a fat deposit was evident in the liver both morphologically (Figure S4A–D) and histologically (Figure 4A–D). As detected by hematoxylin-eosin (HE) staining, large vacuoles of fat were observed in the liver tissues of *Lepr^−/−^* rats, indicating that a fatty liver had developed. Additionally, in the LWE-treated *Lepr^−/−^* group, we observed more normal hepatic cells than those of *Lepr^−/−^* rats (Figure 4D). Similarly, oil red O staining showed that LWE supplementation protected against hepatic steatosis in *Lepr^−/−^* rats (Figure 4E–H). Figure 4K, L shows serum ALT and AST activities, which are biochemical indicators of hepatic damage. The *Lepr^−/−^* rats exhibited remarkably higher ALT and AST levels in comparison with the WT rats, as would be expected. However, compared to the *Lepr^−/−^* control group, consuming LWE reduced the activities of ALT and AST in *Lepr^−/−^* rats by 55.42% and 41.68% (*p* < 0.05), respectively. These results demonstrated that consuming LWE remarkably alleviated fatty liver and protected hepatic injury in *Lepr^−/−^* rats.

### 3.6. LWE Suppressed Liver Lipogenesis in Lepr^−/−^ Rats

**Figure 5:**
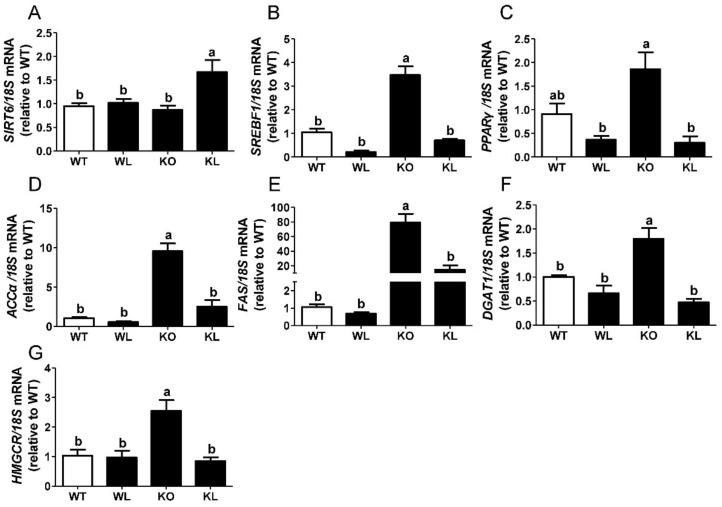
LWE decreased hepatic lipogenic gene expression in the liver of *Lepr^−/−^* rats. (**A**) *SIRT6*; (**B**) *SREBF1*; (**C**) *PPARγ*; (**D**) *ACCα*; (**E**) *FAS*; (**F**) *DGAT1*; (**G**) *HMGCR*. Values are means ± SEM (n = 4–6). Columns with different letters are significantly different (*p* < 0.05).

**Figure 6:**
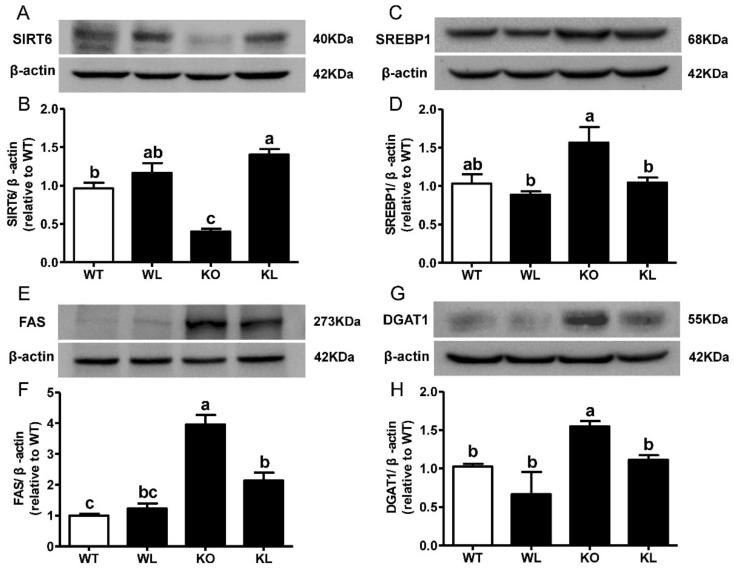
LWE decreased hepatic lipogenic related protein expression in the *Lepr^−/−^* rats. (**A**–**H**) Representative figure and summarized data for SIRT6, SREBP1, FAS and DGAT1. Values are means ± SEM (n = 3–4). Columns with different letters are significantly different (*p* < 0.05).

To explore the mechanisms of LWE actions in alleviating MetS, the expression of genes involved in lipogenesis was analyzed. We observed a remarkable increase in sterol regulatory element-binding transcription factor 1 (*SREBF1*), peroxisome proliferator-activated receptor γ (*PPARγ*), acetyl-CoA carboxylase α (*ACCα*), fatty acid synthase (*FAS*), and diacylglycerol-O-Acyltransferase 1 (*DGAT1*) in the livers of *Lepr^−/−^* rats (*p* < 0.05) (Figure 5A–E). The oral administration of LWE significantly reduced the expression of these adipogenesis genes (all *p* < 0.05). Additionally, the expression of 3-hydroxy3-methylglutaryl-CoA reductase (*HMGCR*) in the livers of *Lepr^−/−^* rats, which encodes the rate-limiting enzyme for cholesterol synthesis, significantly decreased after LWE supplementation (*p* < 0.05) (Figure 5F). Sirtuin 6 (SIRT6) is an NAD^+^-dependent deacetylase implicated in hepatic glucose and lipid metabolism. Our data showed that LWE administration significantly increased the expression of *SIRT6* (Figure 5G). We further analyzed the expression of the proteins controlling lipogenesis in the liver. As shown in Figure 6A, the expression levels of SIRT6 in *Lepr^−/−^* rats were obviously lower than that of WT rats. However, LWE administration obviously increased the expression of SIRT6 in the *Lepr^−/−^* rats. Furthermore, the expression levels of SREBP1, FAS, and DGAT1, which are key enzymes controlling lipogenesis in the liver, were significantly higher in the *Lepr^−/−^* rats than the WT rats, which indicated that liver lipid synthesis was significantly enhanced in *Lepr^−/−^* rats in comparison with WT rats. In contrast, the *Lepr^−/−^* rats with LWE intervention showed a significant reduction in the protein expression levels of SREBP1, FAS, and DGAT1 (Figure 6B–D). The results suggested that LWE intervention prevents the formation of fatty livers and improves serum lipid profiles by suppressing lipid synthesis and accumulation in livers of *Lepr^−/−^* rats.

### 3.7. LWE Increased the Diversity of Gut Microbiota in Lepr^−/−^ Rats

**Figure 7:**
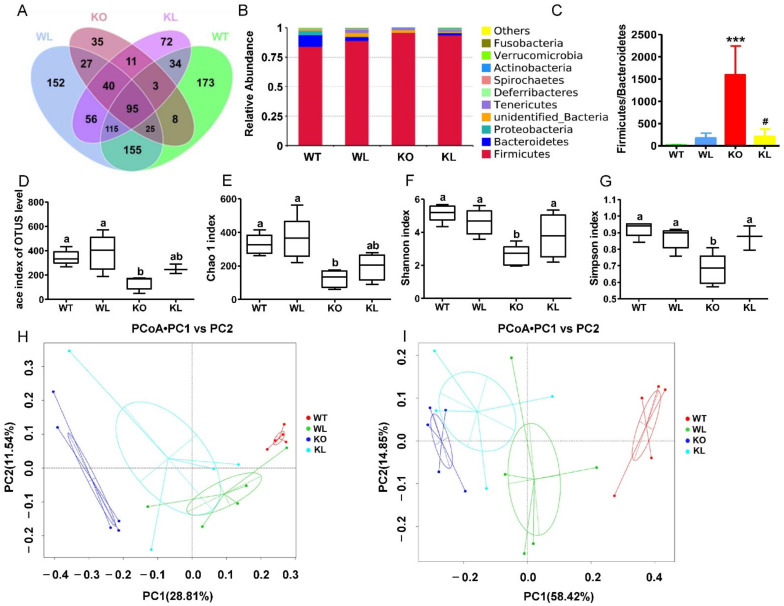
LWE altered the composition and increased the diversity of microbiota in *Lepr^−/−^* rats. (**A**) Venn diagram of gut microbiota in different group rats; (**B**) the abundance of the top 10 Phylum among the different group rats; (**C**) the ratio of relative abundance of *Firmicutes* to *Bacteroidetes*; the alpha diversity of the gut microbiota with different indices: *** *p* < 0.001 when compared to WT group; # *p* < 0.05 when compared to KO group. (**D**) ace index, (**E**) Chao 1 index, (**F**) Shannon index, and (**G**) Simpson index; (**H**) Unweighted UniFrac PCoA (principal coordinates analysis) of gut microbiota; (**I**) Weighted UniFrac PCoA of gut microbiota. Values are means ± SEM (n = 4–5). Columns with different letters are significantly different (*p* < 0.05).

Using the PacBio RS II platform, we performed 16S rRNA sequencing on gut microbiota composition to evaluate the effects of LWE. In total, 1001 bacterial OTUs were collected, and 173, 152, and 72 OTUs were specifically identified in the WT group, WT+LWE group, and *Lepr^−/−^*+LWE groups, respectively, while 35 OTUs were found only in the *Lepr^−/−^* group (Figure 7A). Phylum-level histograms of gut microbiota revealed species composition and their relative abundance (Figure 7B). *Firmicutes* and *Bacteroidetes* made up the vast majority of bacteria (95–96% together) in the microbiota. At the phylum level, the abundance of *Firmicutes* was remarkably increased in *Lepr^−/−^* rats in comparison with WT rats, while LWE supplementation decreased the abundance of *Firmicutes* in *Lepr^−/−^* rats. LWE also decreased the ratio of *Firmicutes* to *Bacteroidetes* (F/B) (Figure 7C).

An analysis of changes in bacterial richness (measured by the Ace and Chao 1 index) and diversity (measured by the Shannon and Simpson index) was conducted following LWE intervention (Figure 7D–G). There was a significantly lower Ace and Chao 1 index in the *Lepr^−/−^* group than in the WT group, indicating that *Lepr^−/−^* rats decreased the richness of the bacterial community. (*p* < 0.05). However, the supplementation of LWE resulted in a higher community richness than in *Lepr^−/−^* groups. Additionally, the Shannon and Simpson index was found to be significantly lower in the *Lepr^−/−^* group than in the WT group. In spite of this, LWE intervention significantly increased the Shannon and Simpson index. In general, LWE treatment significantly increased the α-diversity of gut microbiota in comparison with the *Lepr^−/−^* control group rats. According to the PCoA based on the unweighted and weighted UniFrac statistics, the gut microbiota of *Lepr^−/−^* with LWE groups differed significantly from the *Lepr^−/−^* control groups (Figure 7H,I).

### 3.8. LWE Increased the Microbiota Producing Short Chain Fatty Acids at Genus Level in Lepr^−/−^ Rats

**Figure 8:**
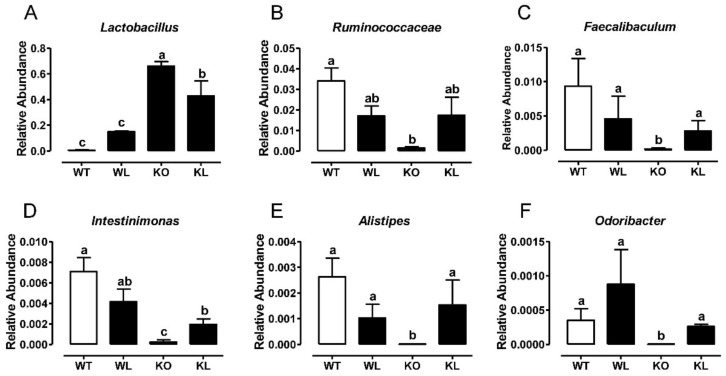
LWE increased the relative abundance of microbiota producing SCFAs in *Lepr^−/−^* rats. Relative abundance of (**A**) *Lactobacillus*, (**B**) *Ruminococcaceae*, (**C**) *Faecalibaculum*, (**D**) *Intestinimonas,* (**E**) *Alistipes*, (**F**) *Odoribacter*. Values are means ± SEM (n = 4–5). Columns with different letters are significantly different (*p* < 0.05).

**Figure 9:**
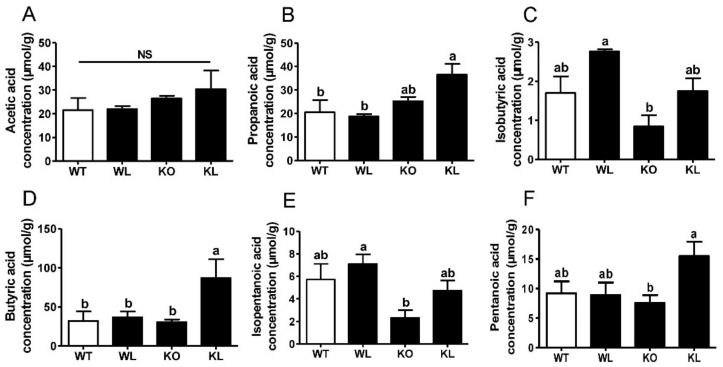
LWE increased the level of SCFAs in *Lepr^−/−^* rats. The concentration of (**A**) acetic acid, (**B**) propanoic acid, (**C**) isobutyric acid, (**D**) butyric acid, (**E**) isopentanoic acid and (**F**) pentanoic acid were measured. Values are means ± SEM (n = 4–5). Columns with different letters are significantly different (*p* < 0.05).

The relative abundance of 30 differential genera was identified and shown in Figure S5. At the genus level, the abundance of *Lactobacillus* was obviously increased, whereas the abundances of *Ruminococcaceae*, *Faecalibaculum*, *Intestinimonas*, *Alistipes,* and *Odoribacter* were dramatically decreased in the *Lepr^−/−^* control group in comparison with WT group (*p* < 0.01) (Figure 8A–F). LWE intervention reversed all these trends in the *Lepr^−/−^* rats. Furthermore, compared with the *Lepr^−/−^* control group, LWE intervention obviously increased the relative abundances of *Akkermansia* and *Veillonellaceae*. Additionally, the majority of gut microbiota altered by LWE were collected to produce SCFAs. Consequently, we detected the effects of LWE intervention on short-chain fatty acid levels in the feces of rats. The results showed that the fecal propanoic acid, butyric acid, isobutyric acid, pentanoic acid, and isopentanoic acid levels were significantly increased after LWE intervention in the *Lepr^−/−^* rats (Figure 9). The results showed that LWE intervention significantly increases the level of SCFAs in *Lepr^−/−^* rats, and these changes might be associated with LWE-modulated specific gut microbiota producing SCFAs.

### 3.9. Amelioration of Metabolic Syndrome Is Associated with the Modulation of Microbiota in Lepr^−/−^ Rats

**Figure 10:**
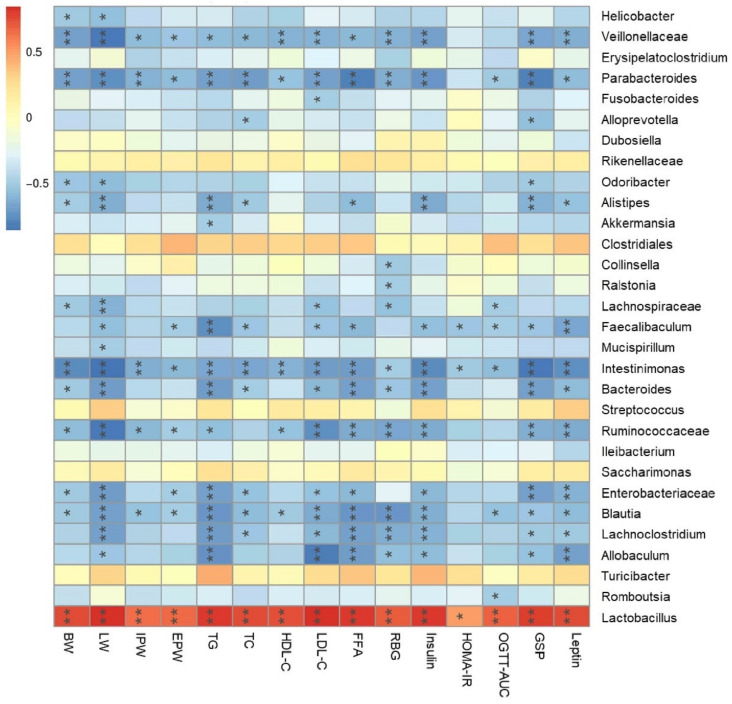
Heatmap of correlation between microbiota and metabolic syndrome parameters. BW, body weight; LW, liver weight; IPW, inguinal pad weight; EPW, epididymal pad weight; RBG, random blood glucose. Significant correlations are denoted as * *p* < 0.05, ** *p* < 0.01.

Spearman’s correlation analysis was used to examine the effects of LWE supplementation on gut microbiota (the relative abundances of 30 differential genera) and specific parameters of the metabolic syndrome across rat groups. The heatmap reflected positive or negative correlations between metabolic syndrome phenotypes and the relative abundances of 30 key genera (Figure 10). The results indicated that the increase of genera in the LWE treated *Lepr^−/−^* groups, which include *Ruminococcaceae*, *Faecalibaculum*, *Intestinimonas*, and *Alistipes*, was strongly negatively correlated with all phenotypes of metabolic syndrome. The abundance of *Odoribacter* was significantly negatively associated with the body weight and glycosylated serum protein. The abundance of *Akkermansia* was only negatively associated with the triglycerides. Moreover, lower abundance genera, such as *Veillonellaceae*, *Parabacteroides*, *Bacteroides*, and *Blautia,* were also significantly negatively correlated with metabolic syndrome-related parameters. In addition, *Lactobacillus*, which were decreased after LWE intervention, were significantly positively correlated with metabolic syndrome-related parameters. The results showed that LWE-induced alterations in gut microbiota composition were closely correlated with phenotypes of metabolic syndrome in *Lepr^−/−^* rats.

## 4. Discussion

A previous study showed that dietary supplementation with large yellow tea powder showed preventive effects on diabetes in mice models [21]. However, whether the large yellow tea water extract (LWE) ameliorates metabolic syndrome in *Lepr^−/−^* rats had not been investigated. In particular, the regulatory effect of LWE on gut microbiota associated with MetS in *Lepr^−/−^* rats is still not clear. Furthermore, there are few studies on tea alleviating MetS that use rat models. The *Lepr^−/−^* rats in the Sprague–Dawley background have complemented some of the defects of current rodent models, such as the extreme and short-term hyperglycemia of db/db mice and the delayed onset of glucose intolerance in Zucker rats, which have proven to be appropriate animal model for the study of MetS biomedically [23]. Our data demonstrated that LWE supplementation at a dosage of 700 mg/kg prevented obesity and fatty liver formation and improved glucose tolerance and hyperlipidemia in *Lepr^−/−^* rats. In this study, the intervention of water infusions of tea mimics the drinking habits of tea consumers in China. A dosage of 700 mg/kg LWE supplemented daily to rats is equivalent to 20 g of tea per day being consumed by an adult human. It is estimated that twenty grams of tea is equivalent to six cups of tea. For regular tea consumers, this amount is acceptable.

In this study, dietary supplementation of LWE remarkably reduced body weight, visceral adiposity, and fatty liver in *Lepr^−/−^* rats. Lipid deposition in the visceral regions is associated with systemic inflammation, insulin resistance, and increased adiposity [36]. According to our results, LWE intervention decreased visceral fat accumulation and adipocyte size, with subsequent improvements in impaired glucose tolerance and insulin sensitivity. The liver is a vital organ that regulates glucose and lipid metabolism as well as energy balance [37,38]. A disorder of hepatic lipid metabolic processes may lead to hepatic steatosis, or steatosis hepatitis [32]. Our data proved that LWE obviously reduces hepatic lipids, including TG, TC, and LDL-C, in *Lepr^−/−^* rats to alleviate fatty liver formation. The results are consistent with a report that found that large yellow tea remarkably reduced fat accumulation in mice fed with a high-fat diet [20].

In our study, the expression levels of *SREBF1*, *PPARγ*, *ACCα*, *FAS,* and *DGAT1* were effectively suppressed via the supplementation of LWE in *Lepr^−/−^* rats. Moreover, the expression levels of SREBP1, FAS, and DGAT1 proteins in liver tissues were also significantly reduced in *Lepr^−/−^* rats via LWE intervention. These results indicated that the suppression of the hepatic lipogenesis pathway may play an important role in mediating the improvement of MetS and hepatic steatosis in *Lepr^−/−^* rats via the supplementation of LWE. Previous research reported that obesity had been prevented by suppressing hepatic lipogenesis [12,39]. SREBP1 is a vital transcriptional factor that regulates lipid and glucose metabolism and targets lipogenic genes coding for *ACCα* and *FAS* [40,41]. Plenty of studies suggest that an increase in SREBP1 levels in a fatty liver may contribute to an increased expression of lipogenic genes, which could lead to increased fatty acid synthesis and increased triglyceride levels [42,43]. Additionally, PPARγ is a nuclear receptor that regulates triglyceride homeostasis, which stimulates the expression of lipogenic genes, contributing to fatty liver formation disease [44,45]. DGAT1 is the rate-limiting enzyme in triglyceride synthesis [46]. Previous research reported that DGAT1 knockout mice exhibit a variety of beneficial characteristics, including improved insulin sensitivity and increased energy expenditure [47]. Therefore, DGAT1 has been considered to be a potential target for treating T2D and dyslipidemia [48].

SIRT6 is an NAD^+^-dependent deacetylase regulating hepatic glucose and lipid metabolism [24]. Cai et al. reported that theaflavins decreased the expression of SIRT6 to reduce lipid accumulation [49]. Khan et al. confirmed that SIRTP6 regulates fatty acid transport by inhibiting PPARγ [50]. Additionally, it has been reported that SIRT6 can suppress SREBP1 to reduce hepatic lipogenesis [29]. In this study, LWE supplementation induced an increase in SIRT6 mRNA and protein expression levels in *Lepr^−/−^* rats. Therefore, increasing the expression of SIRT6 and suppressing the expression of SREBP-1/FAS/DGAT1 may be part of the mechanism of LWE-alleviated MetS and hepatic steatosis in *Lepr^−/−^* rats. Altogether, our results indicated that the improvement of MetS and hepatic steatosis by LWE could be attributed to the activation of the SIRT6/SREBP1 /FAS/DGAT1 pathway to reduce lipogenesis.

Growing evidence indicates that metabolic disorders are closely associated with dysbiosis of the gut microbiota. During this study, LWE supplementation remarkably increased α-diversity and reshaped gut microbiota composition. A previous study reported that obese individuals exhibit approximately 20% more *Firmicutes* and 90% fewer *Bacteroidetes* in comparison to lean individuals [51,52]. In contrast to *Bacteroidetes*, *Firmicutes* metabolized sugar more efficiently, and it was favorable to energy resorption. Thus, higher ratios of *Firmicutes* to *Bacteroidetes* may predict more calorie intake and overweightness [52]. This suggests that LWE treatment has the potential to improve obesity and insulin resistance in *Lepr^−/−^* rats through a decreased ratio of *Firmicutes* to *Bacteroidetes*. Liu et al. reported that the supplementation of green tea, oolong tea, and black tea substantially increases bacterial diversity and alters its structure in obese mice [18]. Chen et al. also reported that kudingcha and fuzhuan brick tea enhanced the microbial diversity of mice [19]. At the genus level of microbiota, the supplementation of LWE prevented the decreased abundance of *Ruminococcaceae, Faecalibaculum, Intestinimonas, Alistipes*, *Odoribacter*, *Akkermansiam* and *Veillonellaceae,* which significantly decreased in *Lepr^−/−^* rats. *Ruminococcaceae*, *Faecalibaculum*, *Intestinimonas*, *Alistipes,* and *Odoribacter* were reported to have the ability to produce short-chain fatty acids. *Ruminococcaceae* was also reported to enhance immune defense and protect against gastrointestinal dysfunction. Published papers reported that a *Ruminococcaceae* abundance was drastically reduced in diabetic or obese rats [53,54]. Moreover, *Ruminococcaceae* are able to enrich bile acid, acetic acid, and butyric acids [55]. In our study, *Ruminococcaceae* were remarkably increased in *Lepr^−/−^* rats via LWE intervention. In addition, *Faecalibaculum* is a group of gram-positive obligate anaerobe that can produce short-chain fatty acids, more specifically butyrate, through fermentation [56,57]. *Intestinimonas* is a representative genus producing butyrate [58]. *Alistipes* is a *Bacteroidetes* species belonging to the *Rickenellaceae* family. It has been demonstrated that obesity and *Intestinimonas* and *Alistipes* abundance are negatively correlated [59]. *Odoribacter* is a butyrate-producing genus in the human gut. A study has revealed that resveratrol treatment improved glucose homeostasis by increasing the abundance of *Odoribacter* [60,61]. Chen et al. found that the proportions of *Alistipes* and *Odoribacter* in high-fat-diet mice were enriched by black tea consumption [19]. Studies suggest that there are important roles for SCFAs in energy metabolism, glucose and lipid homeostasis, and the prevention of metabolic syndrome [62,63,64]. Our results revealed that the fecal propanoic acid, butyric acid, isobutyric acid, pentanoic acid, and isopentanoic acid levels were significantly increased after LWE intervention in *Lepr^−/−^* rats. Furthermore, *Akkermansia* is a bacterium in the *Verrucomicrobia* phylum that is commonly found in the gastrointestinal tract of humans [65,66]. Currently, it has been discovered that *Akkermansia* is a type of probiotic, whose decrease is associated with obesity-related metabolic syndrome. According to a published paper, *Akkermansia* utilize mucins as a source of energy to stimulate goblet cells to produce mucus, which enhances intestinal barrier integrity [67]. Researchers have found that cranberry extract and green tea increase the abundance of *Akkermansia* in mice guts [66,68]. Our data showed that LWE intervention increases *Akkermansia* populations, which might contribute to gut permeability prevention. *Veillonellaceae* constitute a group of bacteria that produce propionate by utilizing lactate as their substrate, and they have a close association with chronic liver disease [69,70,71]. Furthermore, *Parabacteroides*, *Bacteroides*, and *Blautia* were reported to have the ability to produce short-chain fatty acids and were shown to be negatively correlated with the phenotypes of metabolic syndrome [61,72,73]. In addition, our data showed that LWE intervention decreased the abundance of *Lactobacillus* in *Lepr^−/−^* rats. A previous study reported that *Lactobacillus* deferentially attenuates high-fat-induced obesity [74]. However, our study found a rich abundance of *Lactobacillus* in *Lepr^−/−^* rats. Therefore, the effect of *Lactobacillus* on obesity needs further investigation. We propose that the rich abundance of *Lactobacillus* might contribute to the decrease of the other OTUs, and consequently negatively affect the diversity of gut microbiota. Taken together, our results found that LWE treatment increases the SCFA-producing genus and other anti-obesity gut microbiota, which together may exert a beneficial effect on the prevention of MetS and obesity in *Lepr^−/−^* rats. However, the exact mechanism of LWE modulating gut microbiota and anti-obesity needs to be further explored. 

We initially investigated the characteristic components of LWE. The quantitative analysis showed that LWE contains high levels of catechins, and the predominant form of tea catechin is EGCG. Moreover, LWE is also rich in caffeine and polysaccharides. Numerous studies have reported that EGCG and caffeine are important bioactive constituents of tea for reducing body weight and suppressing fat accumulation [75,76,77,78]. Studies have also shown that tea polysaccharides can decrease the risk of T2D, obesity, and other metabolic diseases [79,80]. Thus, we speculated that EGCG, caffeine, and polysaccharides may be the functional components of LWE that ameliorate MetS in *Lepr^−/−^* rats. In addition, the concentrations of GCG in LWE were obviously higher than those of the water extract of Huangshan Maofeng green tea (HWE). Zhou et al. found that the levels of GCG in large yellow tea were enhanced due to the roasting process, and GCG exhibited better inhibitory effects for α-glucosidase than that of EGCG [81]. Therefore, GCG may also contribute to the anti-obesity effects of LWE. However, the mechanisms against obesity complications of the functional components of LWE also need to be further explored.

## 5. Conclusions

In conclusion, our findings suggested that LWE supplementation improved metabolic syndrome in *Lepr^−/−^* rats by modulating gut microbiota composition and suppressing hepatic lipogenesis. The prevention of MetS and fatty liver by LWE is associated with the increase of bacterial diversity and abundance of some bacterial groups producing short-chain fatty acids, such as *Ruminococcaceae*, *Faecalibaculum*, *Intestinimonas*, and *Alistipes*, and the suppression of the SIRT6/SREBP1/FAS/DGAT1 hepatic lipogenesis pathway. These findings offer new perspectives for using dietary LWE as a functional ingredient to prevent metabolic syndrome.

## Figures and Tables

**Table 1:** The contents of catechins, caffeine, amino acids, and polysaccharides in water extract of large yellow tea (LWE) and Huangshan Maofeng green tea (HWE) by UPLC analysis.

Compound	LWE (%)	HWE (%)
EGCG	12.78 ± 0.81	17.81 ± 0.24 **
GCG	6.33 ± 0.09	5.45 ± 0.05 **
EC	5.05 ± 0.56	6.35 ± 0.24
GC	4.45 ± 0.05	4.26 ± 0.04 *
C	2.77 ± 0.04	4.30 ± 0.23 **
Total catechins	31.38 ± 1.44	38.18 ± 0.46 *
Theanine	2.07 ± 0.01	3.05 ± 0.14 ***
Total amino acids	5.03 ± 0.04	7.46 ± 0.56 ***
Caffeine	11.12 ± 0.05	10.69 ± 0.09 *
Polysaccharide	18.80 ± 0.43	15.39 ± 0.14 ***

Abbreviations: (−)-epigallocatechin gallate (EGCG), (−)-gallocatechin gallate (GCG), (−)-epicatechin (EC), (+)-gallocatechin (GC), (+)-catechin (C). LWE, water extract of large yellow tea; HWE, water extract of Huangshan Maofeng green tea. Values are means ± SEM (n = 3). * *p* < 0.05; ** *p* < 0.01; *** *p* < 0.001, compared to the LWE.

## Data Availability

The data presented in this study are available on request from the corresponding author.

## Supplementary Materials

The following supporting information can be downloaded at: https://www.mdpi.com/article/10.3390/foods11111638/s1. Figure S1. Effects of LWE on fat mass ratio and lean mass ratio in each group rats; Figure S2. Effects of LWE on random blood glucose level in each group rats; Figure S3: Effects of LWE on fasting serum glucose, amylin and leptin level in each group; Figure S4: The liver images of different groups of rats; Figure S5: The heatmap of gut microbiota at genus level in different group rats; Figure S6: Total ion current (TIC) chromatograms of short-chain fatty acids in rat feces; Table S1: Compositions of experimental diets; Table S2: Primer sequences used for real-time PCR gene expression experiment.
