# Supplementary material for: Large Yellow Tea Extract Ameliorates Metabolic Syndrome by Suppressing Lipogenesis through SIRT6/SREBP1 Pathway and Modulating Microbiota in Leptin Receptor Knockout Rats
Source: Foods. 2022 Jun 1;11(11):1638. doi: 10.3390/foods11111638 (PMC9180543; doi:10.3390/foods11111638)
Supplement: Supplementary file 1 [file foods-11-01638-s001.zip › foods-1742658-supplementary.pdf]

Supplementary Materials
Figure S1

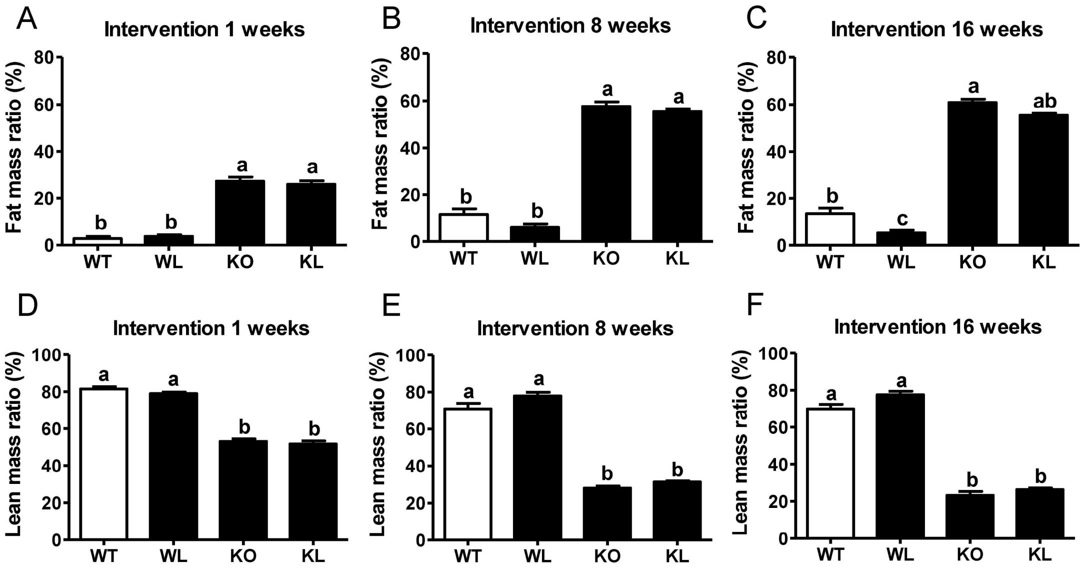

Figure S1. Effects of LWE on fat mass ratio and lean mass ratio in each group rats. Data are expressed as mean ± SEM (n = 4-6). Means with different letters are significantly different ($p < 0.05$).

Figure S2

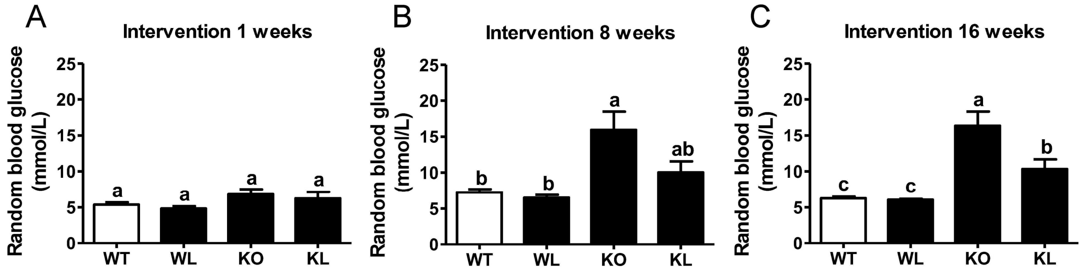

Figure S2. Effects of LWE on random blood glucose level in each group rats. Data are expressed as mean ± SEM (n = 4-6). Means with different letters are significantly different ($p < 0.05$).

Figure S3

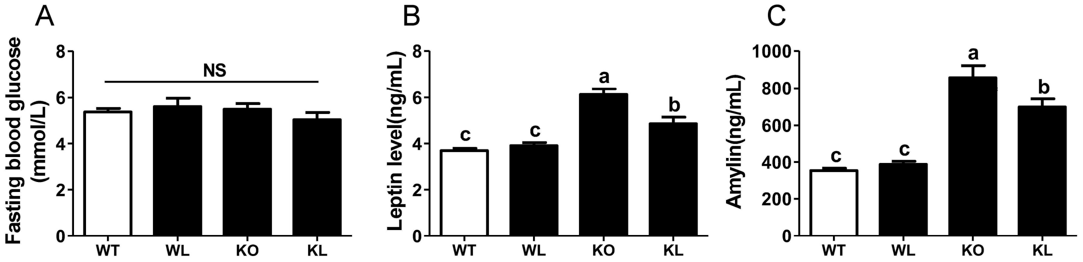

Figure S3. Effects of LWE on fasting serum glucose, leptin and amylin level in each group. (**A**) Fasting serum glucose, (**B**) amylin level and (**C**) leptin level and was measured, respectively. Data are expressed as mean ± SEM (n = 4-6). Means with different letters are significantly different ($p < 0.05$).

Figure S4

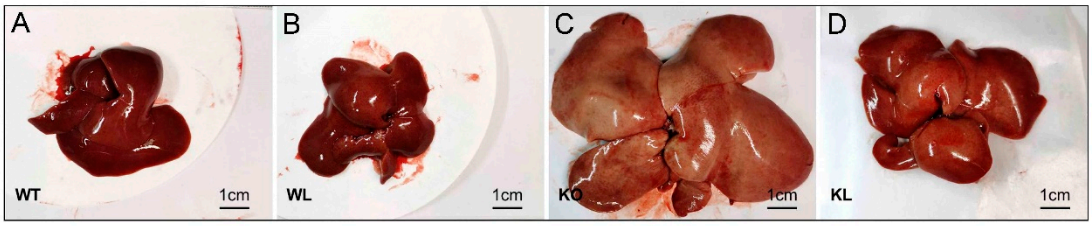

Figure S4. The liver images of different groups of rats.

Figure S5

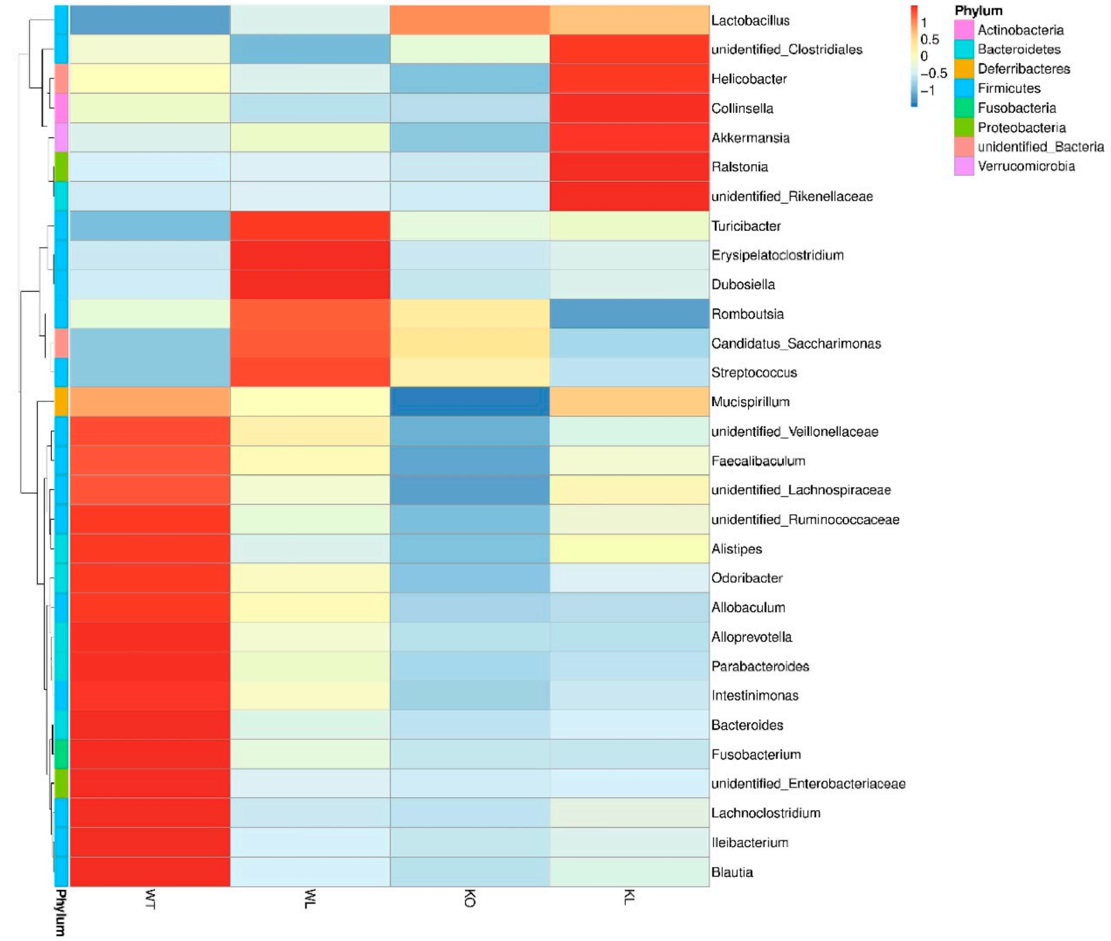

Figure S5. The heatmap of gut microbiota at genus level in different group rats.

Figure S6

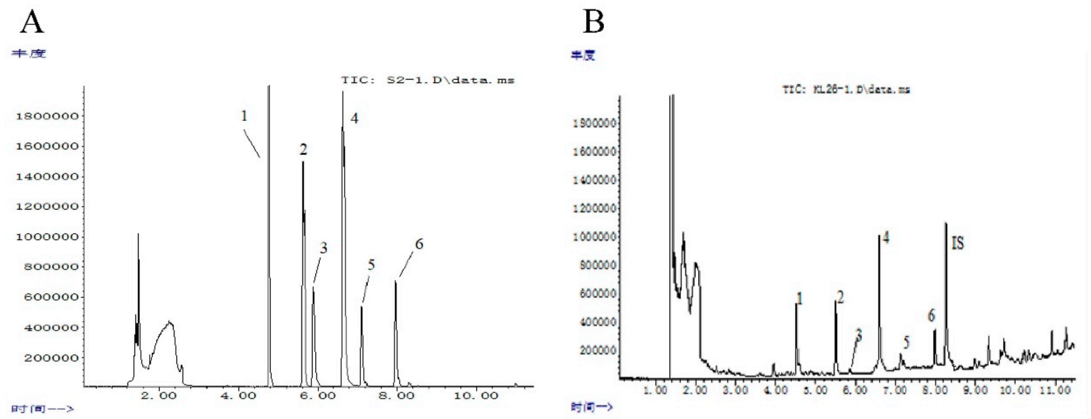

Figure S6. Total ion current (TIC) chromatograms of short chain fatty acids in rat feces. (**A**) total ion current (TIC) chromatograms of standards of short chain fatty acids. (**B**) total ion current (TIC) chromatograms of tested sample of rat feces. The marker in the figure represents 1 acetic acid, 2 propanoic acid, 3 isobutyric acid, 4 butyric acid, 5 isopentanoic acid, 6 pentanoic acid, IS 2-ethylbutyric acid.

Table S1. Compositions of experimental diets

Ingredient (g)	Content percentage
Casein	191
Cornstarch	484
Dextrin	120
Sucrose	66
Soybean oil	24
Lard	19
Cellulose	48
Mineral mix	33
Vitamin mix	10
L-Cystine	3
Line bitartrate	2
TBHQ	0.01
Total	1000
Total energy	
Protein, %	20.6
Fat, %	12.0
Carbohydrate, %	67.4
Energy, Kcal/g	3.616

Table S2. Primer sequences used for real-time PCR gene expression experiment

Gene	Primer sequences<br>Forward (5′-3′)	Reverse (5′-3′)
SREBF1	TCTCCTGGAGCGAGCATTGA	CAGTGGTGGTAGCCATGCTG
PPARγ	GAAAGACAACAGACAAATCACCAT	CAGCTTCCACGGATCGAAACTG
ACCα	GGAAGTGGAAGGCACAGTGAAGG	CTGCGGATCTGCTTGAGGACATAG
FAS	GTTGCTGCTGCTGTGGACCTC	AGGATCACATTGCCGTGGTACTTG
DGAT1	GCCTTACTGGTTGAGTCTATCAC	GCACCACAGATTGACATCC
HMGCR	CCAAACCCAGTAACCCAAAG	GGTAAAACTGCCAGAGAGAAACACT
SIRT6	CGTGGATGAGGTGATGTG	GGCTTATAGGAACCATTGAGA
18S	GGGTCGGGAGTGGGTAATTT	AGAAACGGCTACCACATCCAA

The original unprocessed images for western blot and the target panels were marked in red arrow

Figure 6A

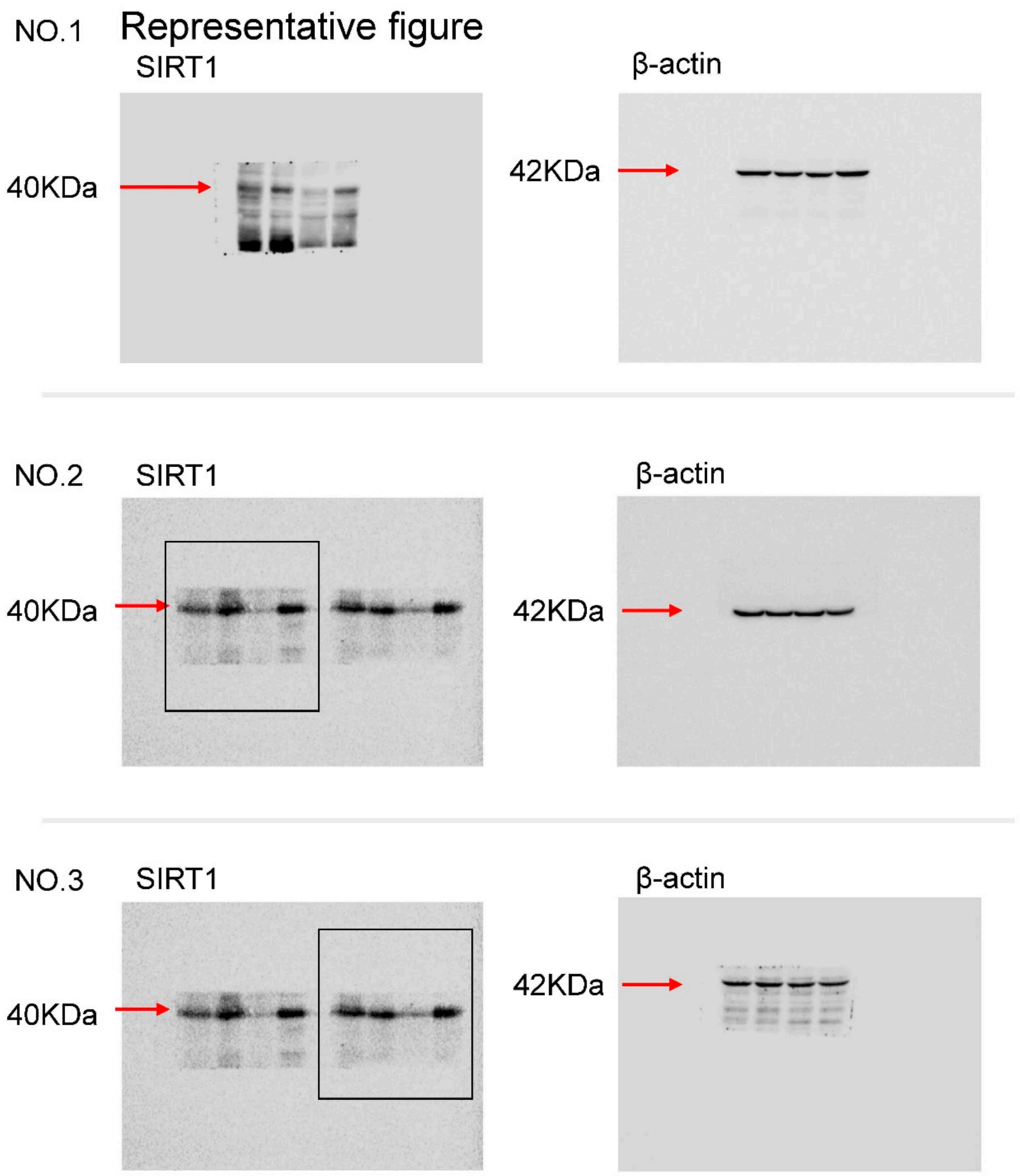

Figure 6B

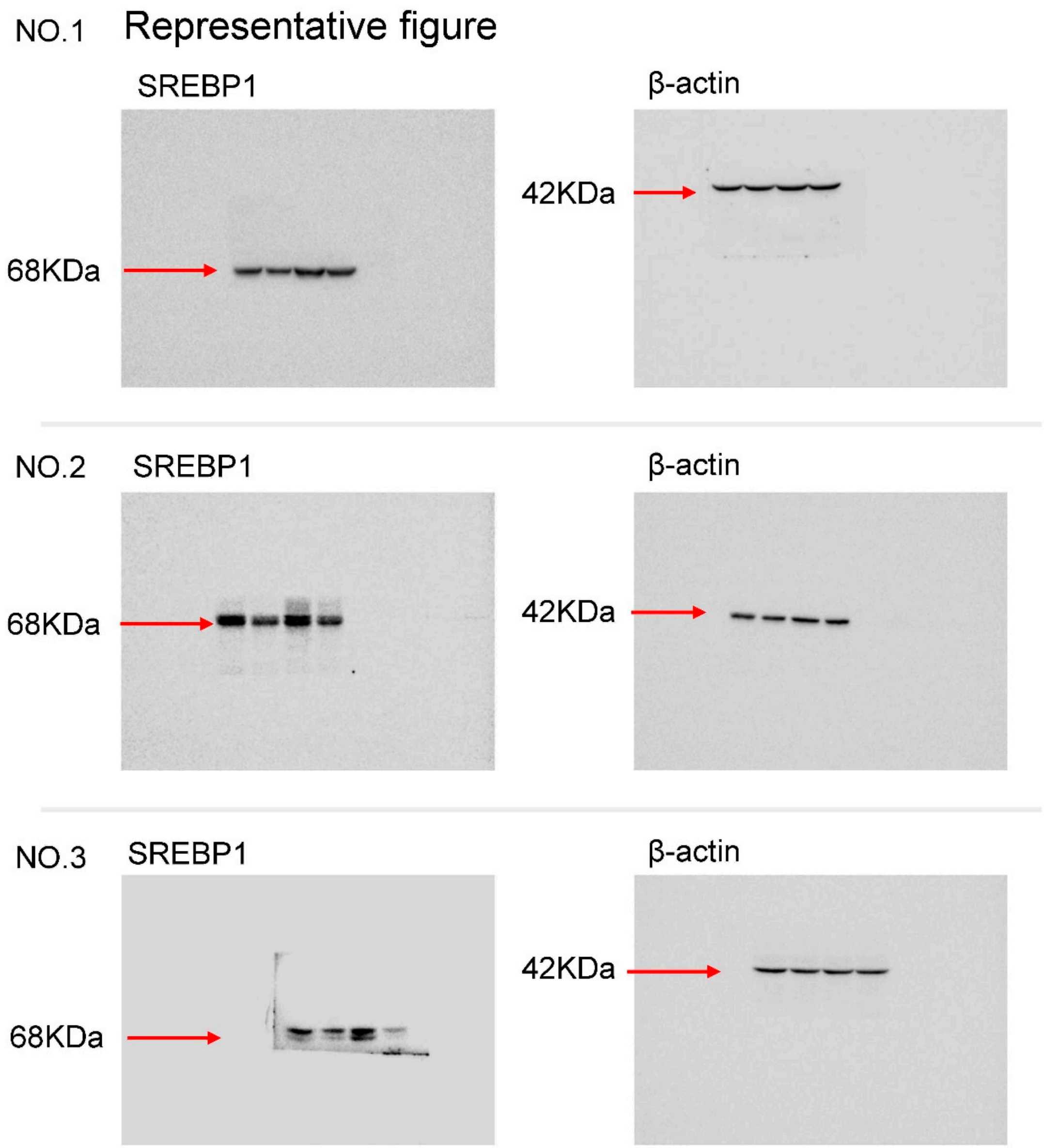

NO.1
Representative figure
SREBP1
β-actin
42KDa
68KDa
NO.2
SREBP1
β-actin
68KDa
42KDa
NO.3
SREBP1
β-actin
42KDa
68KDa

Figure 6C

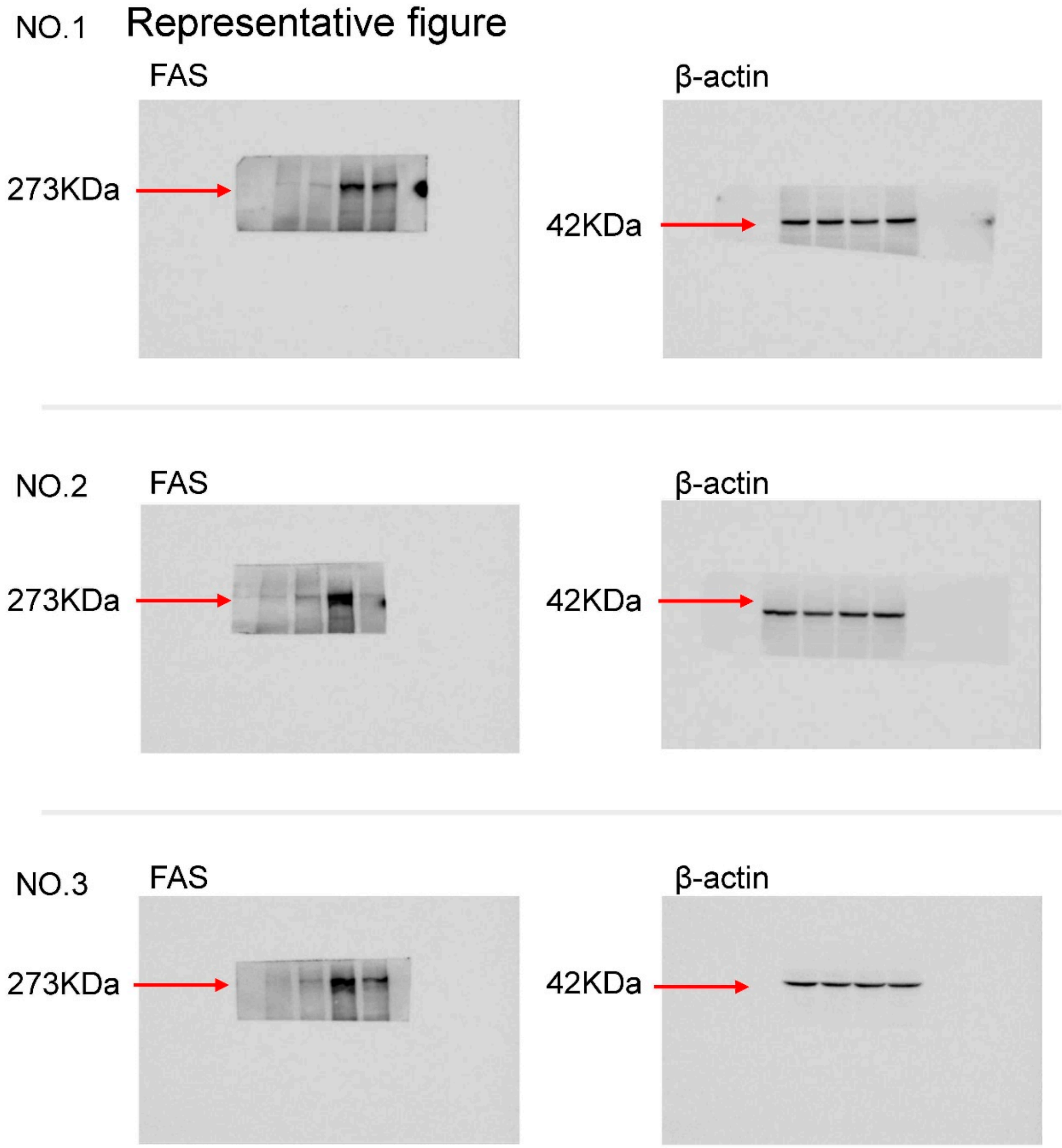
NO.1
Representative figure
FAS
β-actin
273KDa
42KDa
NO.2
FAS
β-actin
273KDa
42KDa
NO.3
FAS
β-actin
273KDa
42KDa

Figure 6D

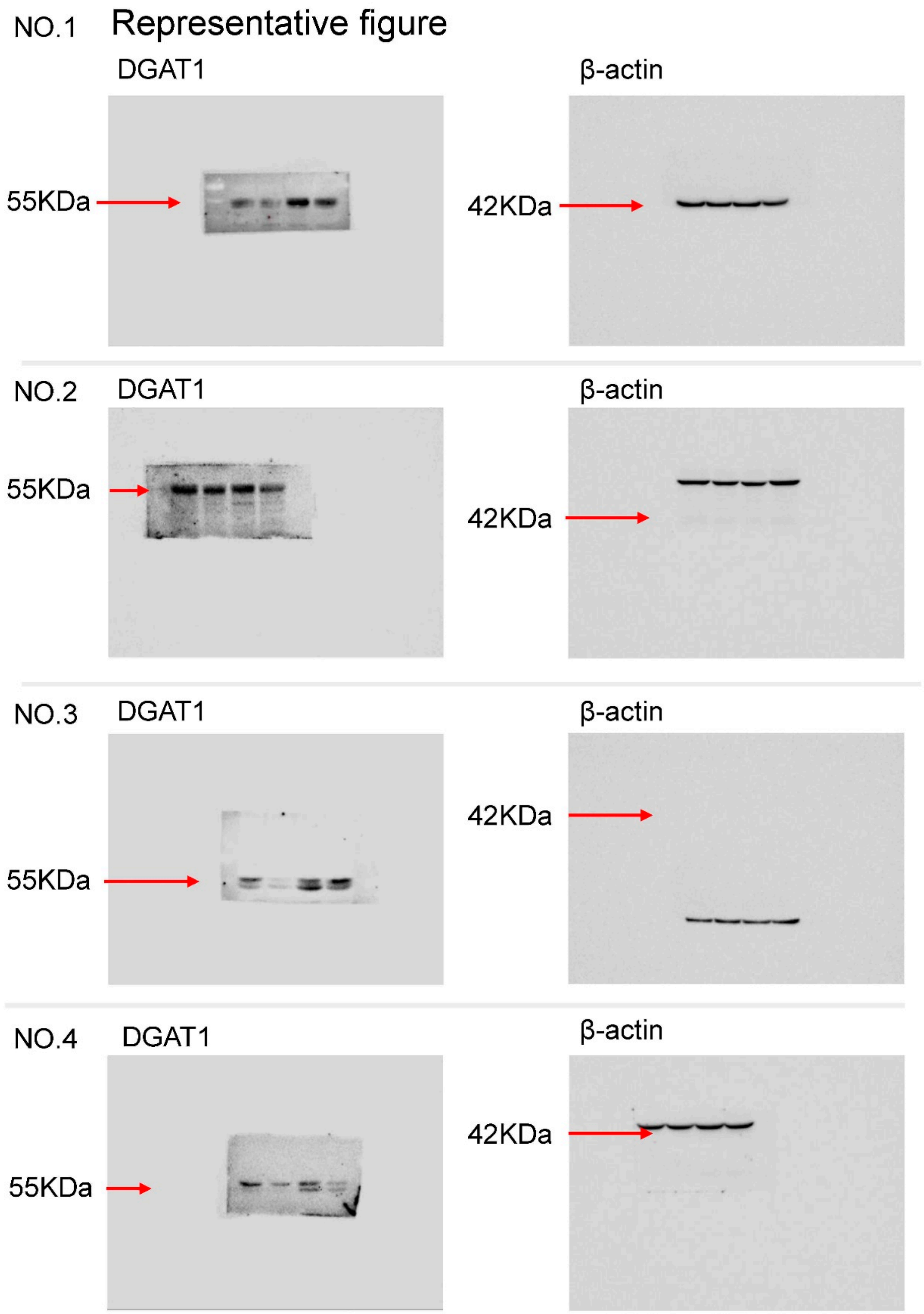
NO.1
Representative figure
DGAT1
β-actin
55KDa
42KDa
NO.2
DGAT1
β-actin
55KDa
42KDa
NO.3
DGAT1
β-actin
42KDa
55KDa
NO.4
DGAT1
β-actin
42KDa
55KDa
